# Supplementary figures and images for: Lower Locus Coeruleus MRI intensity in patients with late-life major depression
Source: PeerJ. 2021 Feb 16;9:e10828. doi: 10.7717/peerj.10828 (PMC7894108; doi:10.7717/peerj.10828)

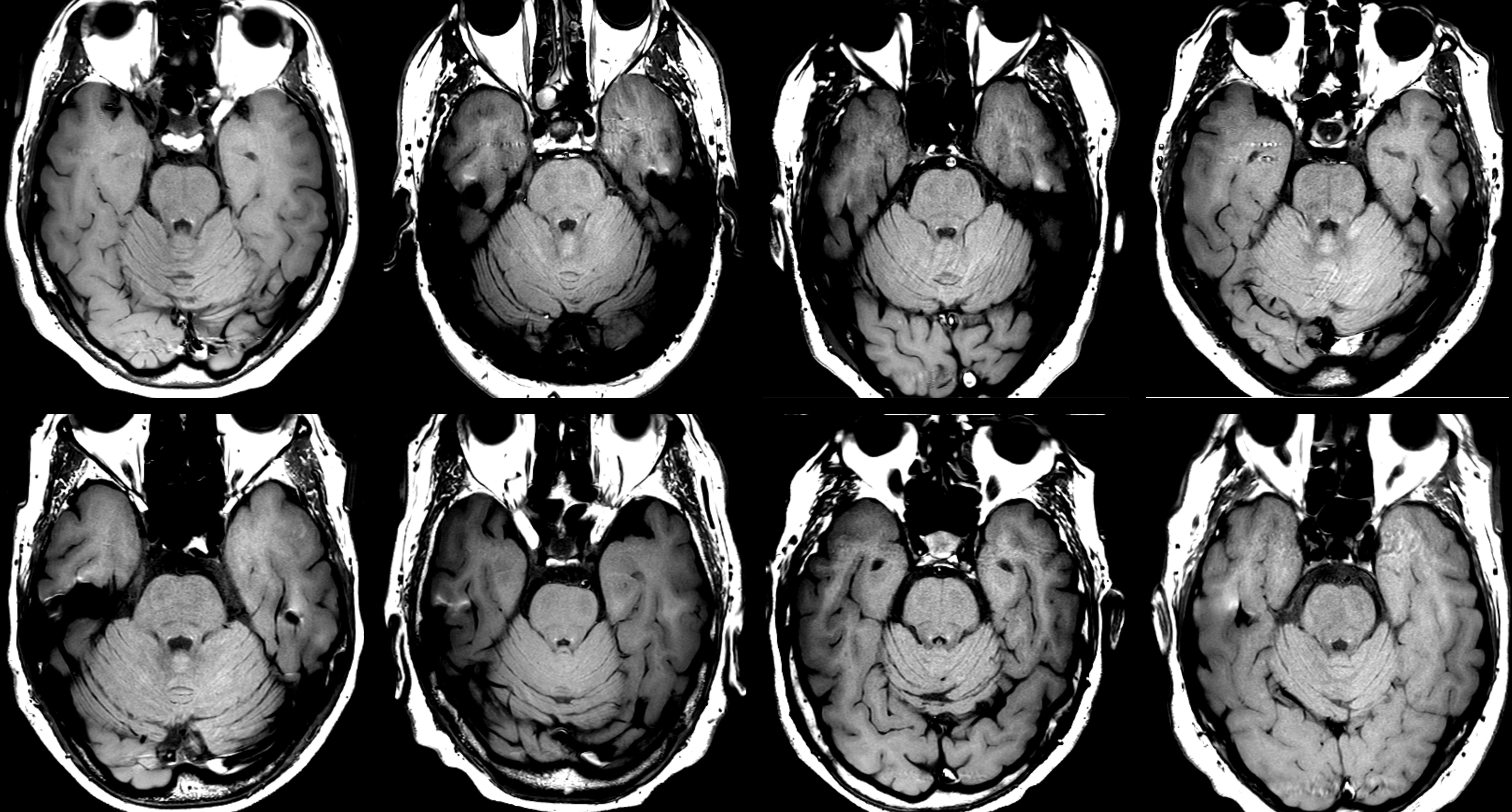

Supplement: Supplemental Information 1 — Axial slices at the level of the pontine tegmentum of eight representative individuals (from the three study groups: 4 HC, 2 patients with late-life MDD and 2 patients with aMCI) from the T1-weighted fast spin-echo sequence used for LC localization. The LC is visible as two symmetric hyperintensities ventral to the fourth ventricle. [file peerj-09-10828-s001.jpg]

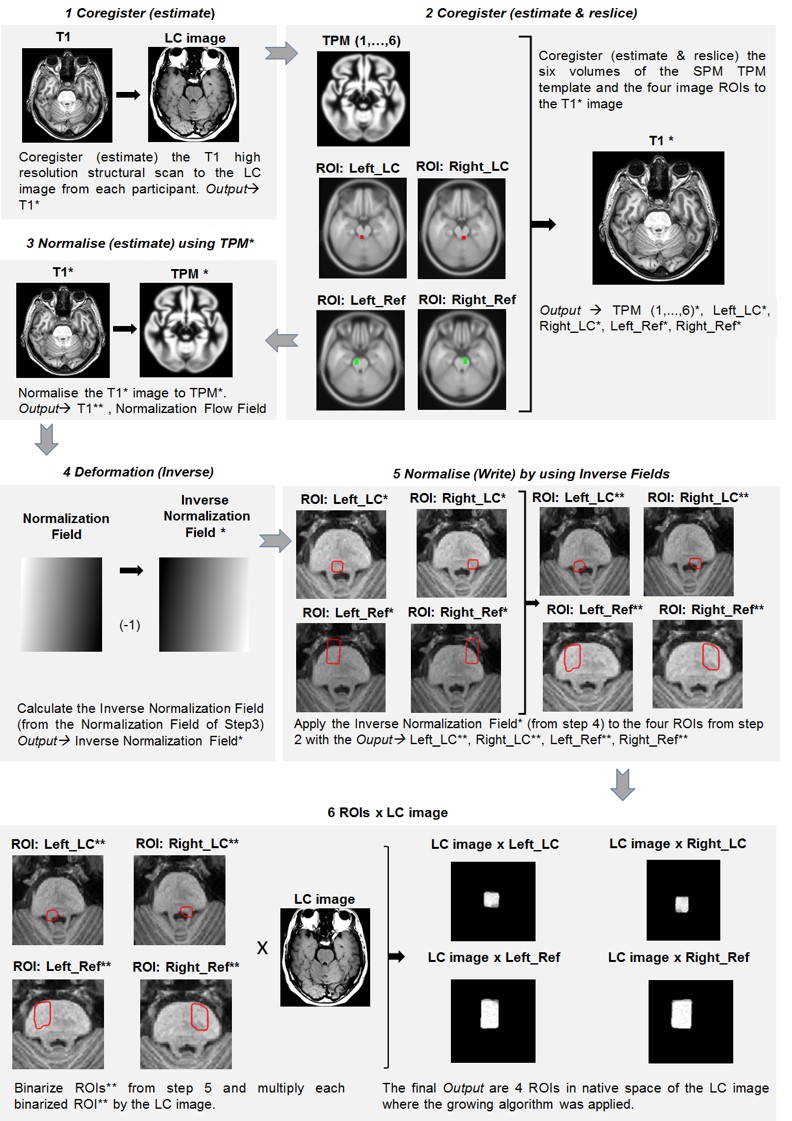

Supplement: Supplemental Information 2 — LC, Locus Coeruleus; ROI, Region of Interest; TPM, tissue probability map. [file peerj-09-10828-s002.jpg]

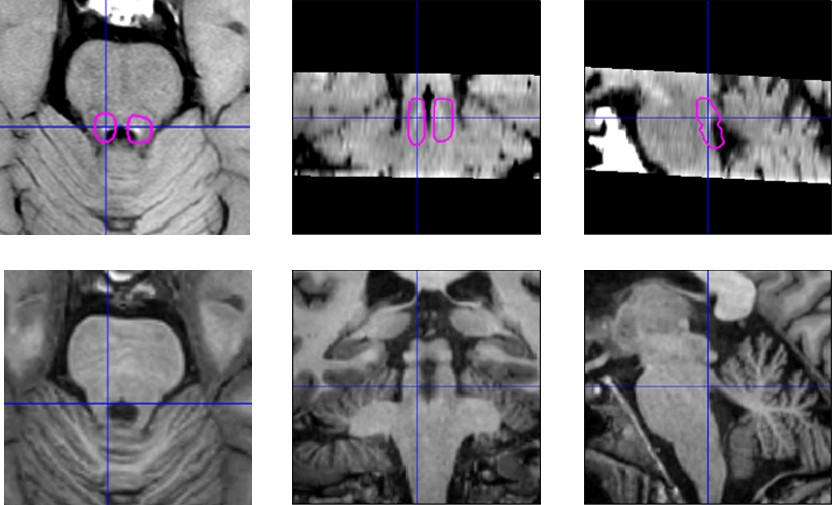

Supplement: Supplemental Information 3 — The denormalized ROIs of the LC area (magenta contours) are overlaid onto axial, coronal and sagittal slices of a native space LC image from one representative participant (top). The same brainstem sections are depicted in the bottom image using slices from the high-resolution whole-brain T1 sequence. The crosshair is depicted to facilitate comparison between the images. [file peerj-09-10828-s003.jpg]

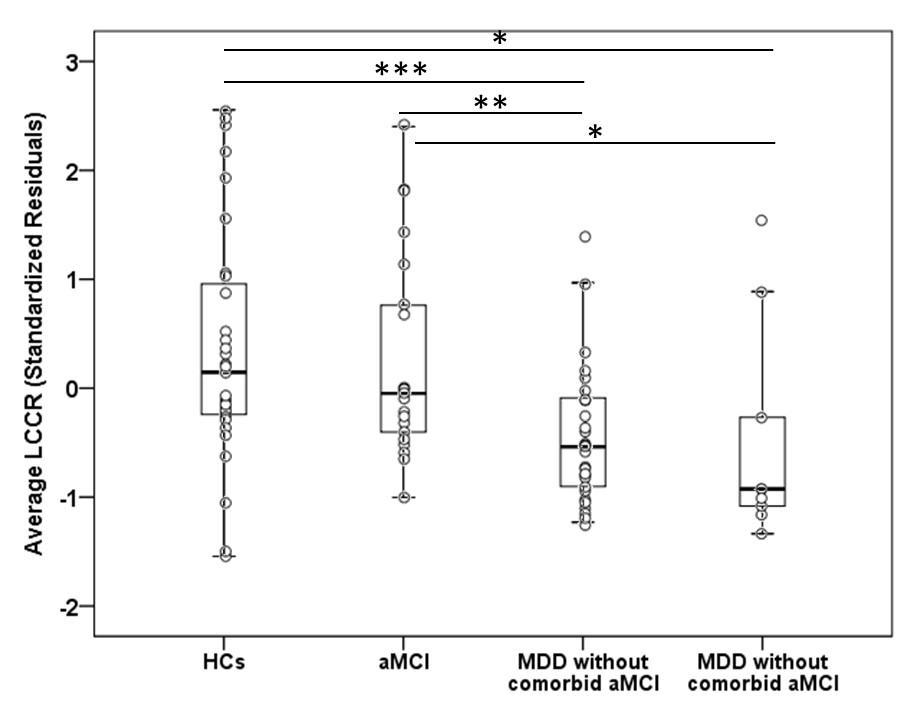

Supplement: Supplemental Information 4 — The individual values (dots) are overlaid for reference. aMCI, amnestic Mild Cognitive Impairment; HCs, Healthy Controls; LCCR, Locus Coeruleus Contrast Ratio; MDD, Major Depressive Disorder. * p ≤ 0.05, ** p ≤ 0.005, *** p ≤ 0.001. All results remained significant after excluding outlier values. [file peerj-09-10828-s004.jpg]

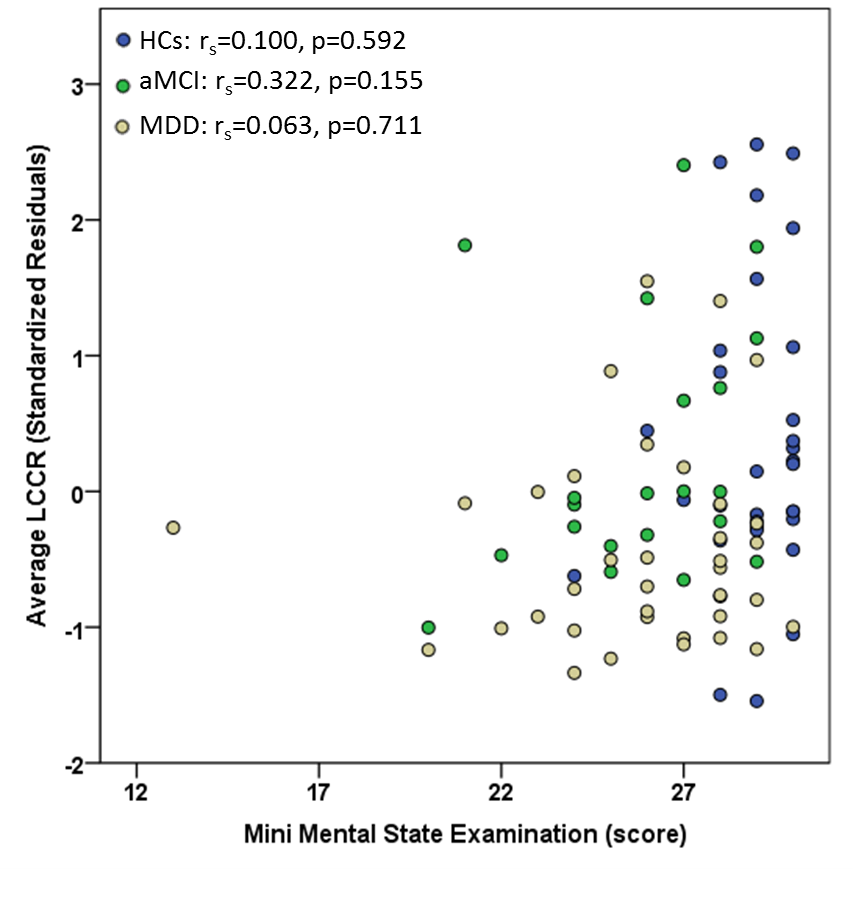

Supplement: Supplemental Information 5 — aMCI, amnestic Mild Cognitive Impairment; HCs, Healthy Controls; LCCR, Locus Coeruleus Contrast Ratio; MDD, Major Depressive Disorder; rs, Spearman’s correlations. [file peerj-09-10828-s005.png]
