# Supplementary material for: Lower Locus Coeruleus MRI intensity in patients with late-life major depression
Source: PeerJ. 2021 Feb 16;9:e10828. doi: 10.7717/peerj.10828 (PMC7894108; doi:10.7717/peerj.10828)
Supplement: Supplemental Information 6 — 1 Years from depression onset till MRI scanning. 2 Thase and Rush Staging of Treatment-Resistant Depression: Thase, M.E.; Rush, A.J. Treatment-resistant depression. In: Bloom, F.E., Kupfer, D.J. (Eds). Psychopharmacology: The Fourth Generation of Progress. New York, NY: Raven Press, 1995, 1081–1098. [file peerj-09-10828-s006.doc]

|  | Hamilton Depression Rating Scale (HDRS) | Geriatric Depression Scale (GDS) | Disease Duration^1^ | Treatment Resistance^2^ |
| --- | --- | --- | --- | --- |
| MDD taking SNRIs (n=25)  Mean (SD) | 11.12 (6.629) | 4.96 (4.238) | 13.48 (8.037) | 1.8 (0.764) |
| MDD not taking SNRIs (n=12)  Mean (SD) | 14.17 (8) | 6.83 (4.152) | 12.92 (8.028) | 1.67 (1.231) |
| Mann–Whitney U test | 117.5 | 105 | 147.5 | 145 |
| Z | -1.058 | -1.473 | -0.081 | -0.170 |
| P-value | 0.290 | 0.141 | 0.935 | 0.865 |
| δ (Cliff’s Delta) | -0.217 | -0.3 | -0.017 | -0.03 |
