# Supplementary material for: Lower Locus Coeruleus MRI intensity in patients with late-life major depression
Source: PeerJ. 2021 Feb 16;9:e10828. doi: 10.7717/peerj.10828 (PMC7894108; doi:10.7717/peerj.10828)
Supplement: Supplemental Information 7 — 1Tricyclics and/or Mirtazapine and/or Vortioxetine and/or Agomelatine and/or Bupropion; LCCR, Locus Coeruleus Contrast Ratio; MDD, Major Depressive Disorder; SD, Standard Deviation; SNRIs, Serotonin and Norepinephrine Reuptake Inhibitors. sraLCCR values of MDD patients taking noradrenergic medication other than SNRIs (n = 6) did not differ from MDD patients taking SNRIs (n = 25) (U = 51, Z = − 1.2, p = 0.23, δ = − 0.32), or from MDD patients not taking noradrenergic medication (n = 6) (U = 17, Z = − 0.16, p = 0.873, δ = − 0.06). Likewise, these patients did not either differ from HCs (U = 66, Z = − 1.11, p = 0.266, δ = − 0.29) or aMCI patients (U = 49, Z = − 0.816, p = 0.414, δ = − 0.23). [file peerj-09-10828-s007.doc]

|  | **Average LCCR**  **Mean (SD)** | ***sra*LCCR**  **Mean (SD)** |
| --- | --- | --- |
| MDD taking SNRIs (n=25) | 0.180 (0.045) | -0.621 (0.538) |
| MDD taking noradrenergic medication other than SNRIs^1^ (n=6) | 0.214 (0.071) | -0.155 (0.908) |
| MDD not taking noradrenergic medication (n=6) | 0.234 (0.085) | -0.032 (1.068) |
